# Review on extraction technology and function of plant-derived exosome-like nanoparticles

**DOI:** 10.3389/fmedt.2025.1668738

**Published:** 2025-11-10

**Authors:** Jiali Cheng, Yi Zhu

**Affiliations:** College of Food Science and Nutrition Engineering, China Agricultural University, Beijing, China

**Keywords:** plant-derived exosome-like nanoparticles, isolation, stability, drug delivery, challenges

## Abstract

**Introduction:**

Plant-derived exosome-like nanoparticles (PELNs) are currently a hot research topic, which have been confirmed to have similar structures and functions to mammalian-derived exosomes. PELNs are lipid bilayer membrane nanovesicles containing bioactive constituents such as miRNA, mRNA, protein, and lipids obtained from plant cells, that can participate in intercellular communication and mediate transboundary communication, have high bioavailability and low immunogenicity, are relatively safe, and have been shown to play an important role in maintaining cell homeostasis and preventing, and treating a variety of diseases.

**Methods:**

The author has read recent articles on PELNs and summarized them.

**Results:**

We summarized the importance and challenges of PELNs and provided a theoretical basis for the future research and clinical application of PELNs.

**Discussion:**

In this review, we describe the biogenesis, isolation and purification methods, structural composition, stability and function of PELNs, mainly introducing the role of PELN in anti-inflammatory, anti-tumor, and drug delivery.

## 1 Introduction

Extracellular vesicles (EVs) refer to a type of heterogeneous particles naturally released into the extracellular matrix by various living cells through different mechanisms (1). It is enveloped by a phospholipid bilayer, containing the cytoplasmic matrix, proteins (enzymes), lipids, nucleic acids (mRNA, miRNA, tRNA, rRNA, and DNA), and bioactive molecules (polysaccharides, pigments, and toxins) of the parent cell (2). The International Society for Extracellular Vesicles (ISEV) collectively refers to “particles that are separated by lipid bilayers, naturally released by cells, and cannot replicate” as “EVs” (3). At present, only mammalian EVs have a relatively detailed classification, which is divided into microvesicles, exosomes, and apoptotic bodies according to different modes of biogenesis (4). Extracellular vesicles are currently a hot research topic, and they appear as cup-shaped, biconcave, or spherical vesicle structures ranging from 30 to 150 nm under transmission electron microscopy (5).

**Figure 1:**
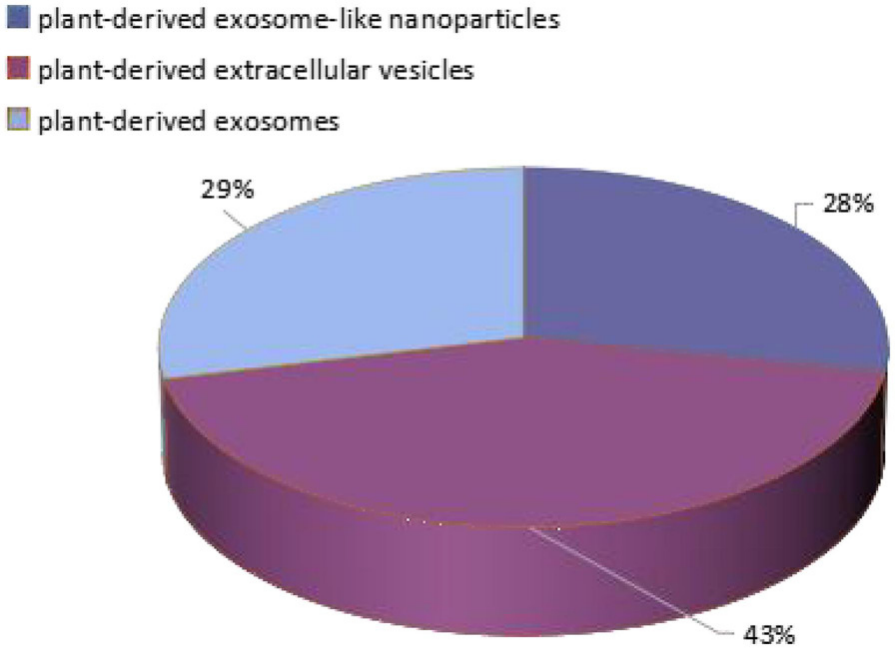
The proportion of three names in the database.

In recent years, vesicles with a structure similar to exosomes have been isolated from dietary plants, and their protein composition is as similar as that of exosomes by up to 50% (6), with a size generally ranging from 30 to 400 nm (7). However, the term ‘exosomes’ has traditionally only been used to describe mammalian EVs, and EVs from various unconventional sources have not yet been systematically classified. In most studies, plant derived nano components are referred to as “plant-derived exosomes”, “plant-derived exosome-like nanoparticles”, or “plant-derived extracellular vesicles”. There is no unified naming system for these terms, so different articles will use different names, which appears chaotic. As shown in Figure 1, three different terms will be searched in all databases, and their proportions can be seen. There are 43% articles named after “plant-derived extracellular vesicles”, 29% named after “plant-derived exosomes”, 28% named after “plant-derived exosome-like nanoparticles”. Therefore, a unified name should be adopted as soon as possible to avoid some disputes. For the convenience of description, this article uses plant-derived exosome-like nanoparticles (PELNs) as a general term. At the same time, the author also calls on researchers to adopt such a unified naming convention to make articles in this research field more professional and consistent in terminology.

In recent years, researchers have isolated, purified, and identified PELNs from various plants such as semen raphani, garlic, ginger, houttuynia cordata, and blueberries. Their structures and chemical compositions have been characterized, and their physiological functions have been evaluated. Compared to animal derived exosomes, PELNs exhibit superior biocompatibility, low immunogenicity, high safety, and green sustainability, making them highly promising in the biomedical field. PELNs, as therapeutic agents, can be used for the treatment of colitis, anti-tumor, and liver diseases. They can also be used as drug delivery carriers. The low immunogenicity and special surface components can promote their entry into organs, tissues, and cells (8), showing broad application prospects. The article carries on the discussion regarding it. This review aims to provide researchers with deeper insights into PELNs, stimulate possible new research ideas, further promote the clinical application of PELNs medical materials, and accelerate innovation and development in the biomedical field. With the deepening of research and technological progress, it is expected that PELNs will play a more important role in the future medical practice field.

## 2 Formation and composition of PELNs

### 2.1 Formation of PELNs

For higher plants, as early as 1965, Jensen (9) observed cotton bead cores using electron microscopy and found that almost all bead cells contained multivesicular bodies (MVBs), some of which were protruding components of the endoplasmic reticulum and some were free in the cytoplasm. Subsequently, in 1967, Halperin & Jensen (10) observed a large number of MVBs clearly derived from the Golgi apparatus in wild-type carrot suspension cultured cells, and found that they may be involved in the material exchange process between the endoplasmic reticulum and the Golgi apparatus. Although PELNs were discovered earlier than mammals, their biological functions have not been uniformly determined for a long time. In Arabidopsis, indole glucosinolates are important defense components during infection, activated by myrosinase, and their formation and transport have been found to be mediated by PELNs (11). In addition, barley and leguminous plants selectively enhance the deposition of callus on the cell wall by secreting PELNs to resist pathogens (11). Therefore, researchers speculate that the production of PELNs contributes to the development of early defense structures (polarization defense and chemical defense).

**Figure 2:**
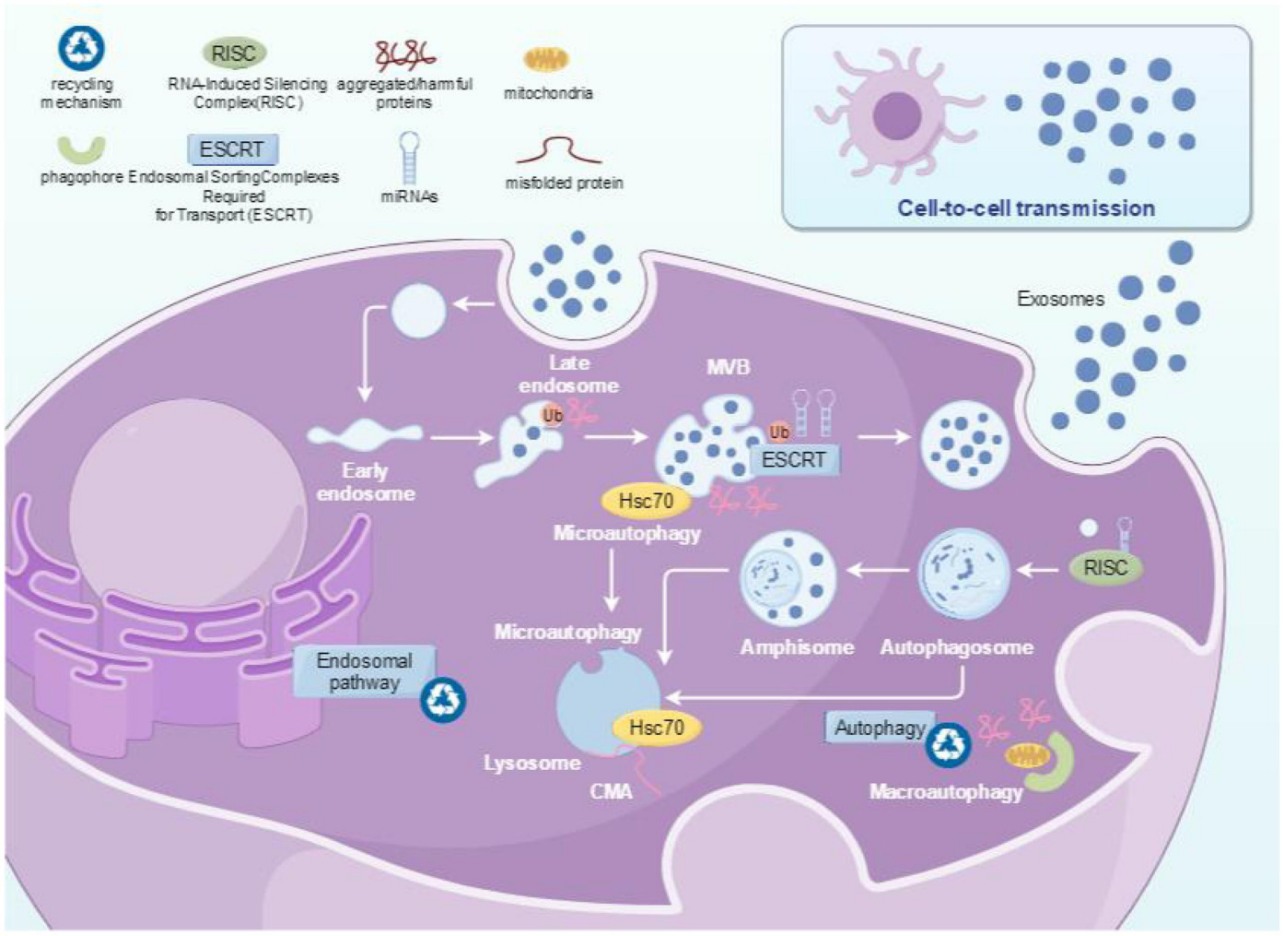
The formation pathway of PELNs.

There are two reported pathways for the formation of extracellular vesicles, one is the “classical pathway” of extracellular vesicle formation (Figure 2), and the other is the direct pathway of extracellular vesicle formation (12). In the “classical pathway”, extracellular vesicles are produced through two membrane invaginations (13, 14): firstly, some extracellular substances, such as proteins, lipids, metabolites, and ions, together with protein molecules on the cell membrane, sink inward through the cytoplasmic membrane to form small vesicles of early endosomes (EE). When the early endosomes mature, the endosome membrane will further sprout inward to form late endosomes (LE), also known as multivesicular bodies (MVBs). Due to the occurrence of two membrane invaginations, intraluminal vesicles (ILVs) are formed. ILVs appeared in this process. Subsequently, MVBs containing ILVs will be transported to the cell membrane and fused with it, releasing ILVs into the extracellular space. The released vesicles are exosomes (15), which are also the pathway for PELNs formation, primarily forming via invagination of the plant plasma membrane (16). In addition, MVB can also fuse with lysosomes inside cells, leading to degradation of vesicle contents. However, the mechanism by which MVB is sorted and fused with the cytoplasmic membrane to produce exosomes or fused with lysosomes to be degraded remains unclear so far. The second pathway is the direct formation of extracellular vesicles, which are released directly from the cytoplasmic membrane (12). The extracellular vesicles formed by this pathway have similar diameter and density to those released by classical pathways, which are commonly found in T cells or erythroleukemia (12). It is currently unclear whether these extracellular vesicles will be released in other cells or body fluids. In addition to the two pathways above, the biogenesis of exosomes in plants can also take an alternative pathway. Double membrane organelles known as EXocyst Positive Organelles (EXPOs) have been identified in some plants like tobacco. They are similar to autophagosomes except that they are not associated with any degradation pathway, instead, they are involved in a direct secretion pathway (17).

### 2.2 Composition of PELNs

**Figure 3:**
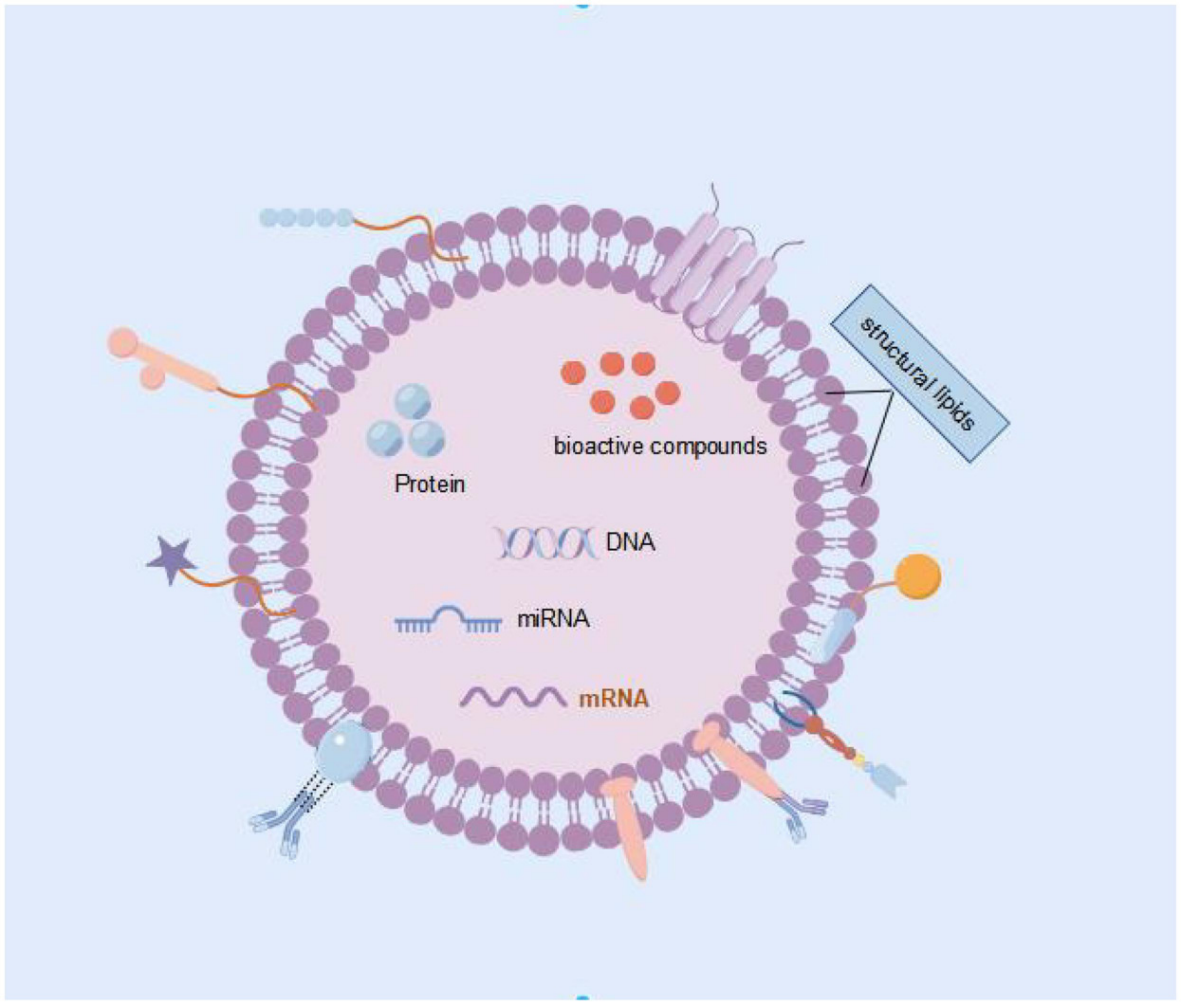
Composition of exosomes.

PELNs have a phospholipid bilayer membrane structure, with a large amount of proteins on the membrane and a large amount of lipids, DNA, and RNA inside the membrane (Figure 3), which can regulate various physiological or pathological reactions (18). Protein analysis shows that PELNs contain proteins involved in signal transduction, many of which are highly induced in stress and immune responses; It also includes proteins involved in reactive oxygen species signaling and oxidative stress response, as well as various membrane transporters and vesicular transporters (19). Lipids are mainly composed of phosphatidic acid (PA), PE, phosphatidylinositol (PI), diacyl glycerol (DAG), triacyl glycerol (TAG), digalactosyldiacylglycerol (DGDG), and monogalactosyldiacylglycerol (MGDG) (20). PELNs contain various RNA components, such as miRNA, sRNA, and other non coding RNAs (7). Recently, 418 conserved miRNAs were identified from 11 edible fruits and vegetables (at least 32 from ginger and at most 127 from soybean) (21).

## 3 Isolation and identification of PELNs

The experience gained from the industrial production of liposomes, monoclonal antibodies, and viruses has strong reference significance for the development of separation methods for PELNs (22). The requirements for separating samples vary for different experimental purposes. ISEV regulations require high concentrations of extracellular vesicles for diagnostic purposes, but the structural integrity of extracellular vesicles used for drug delivery needs to be considered first (23). Even if the separation standards are lowered, a single separation method is often limited by yield, purity, and cost in highly complex biological fluid samples. Nowadays, most studies often use a combination of two or more methods. Our laboratory is also exploring the use of polymer precipitation and size exclusion chromatography to separate and purify extracellular vesicle like nanoparticles from Semen Raphani.

### 3.1 Separation of PELNs

Traditional separation methods are based on the different sizes and densities of vesicles, such as ultracentrifugation (UC), filtration, and size exclusion chromatography (SEC). Subsequently, solubility based methods such as chemical precipitation have emerged. Recently, new methods such as affinity chromatography and microfluidics have been developed based on highly specific interactions between PELNs surface proteins (22). The separation method largely determines which subpopulations of vesicles are obtained, and further determines its applicability for downstream applications (Table 1).

**Table 1:** Comparison of advantages and disadvantages of separation methods.

Separation method	Principle	Advantage	Disadvantage
Ultracentrifugation	Density	“Gold standard”, easy to operate, can separate a large number of samples	Long duration, expensive equipment, and high centrifugal speed may affect the integrity of EVs
Density gradient centrifugation	Density	High separation purity, no other chemical contamination	Long duration (>4 h), large sample size, need for centrifugation, low recovery rate
Ultrafiltration	Size	The program is simple and allows for simultaneous processing of multiple samples, saving time and maintaining EV activity well	Impurities smaller than the pore size of the filter membrane cannot be filtered out, and membrane like structures such as cells need to be removed before using the sample
Size exclusion chromatography	Size and molecular weight	Efficient, easy to operate, and short in time, with high yield and purity of extracted exosomes, which can maintain the integrity of EV	High cost, requiring specialized equipment, limited frequency and sample volume of chromatographic columns, complex protein aggregates, and co separation of lipoproteins and lipoproteins
Polymer precipitation	Surface charge	Easy to operate, fast, and cost-effective, with minimal damage to EVs	The purity of isolated exosomes is usually very low
Immunoaffinity capture-based technique	Surface antigen affinity	High purity, high specificity	Antibody preparation has limitations and high costs
Microfluidics technology	Size, density, and surface antigen affinity	Miniaturization, integration, high throughput, and short time consumption	The research technology has not been standardized yet; High cost and expensive equipment

#### 3.1.1 Ultracentrifugation

UC is the “gold standard” for separating extracellular vesicles, where centrifugal force, time, and rotor type all affect the yield and purity of PELNs (24–26). Firstly, plant sap is extracted by homogenization, crushing, soaking, etc., followed by a series of differential centrifugation to collect the supernatant. Finally, PELNs are obtained by centrifugation at a speed of at least 100,000× g. This step usually involves co enrichment of similar sized impurities such as proteins and lipids, which require further purification. This method is simple to operate and produces a large amount of extracellular vesicles, but the process is time-consuming and the recovery rate is unstable. Repeated centrifugation may cause damage to the extracellular vesicles (23, 27). This method has low separation efficiency for plant samples with high viscosity or high impurity particles, such as kudzu root and yam (7). In addition, the high-speed centrifuge is a large-scale equipment that limits the method to laboratory scale research. Further clinical and industrial production still need to address issues such as low yield, inter batch instability, non standardized procedures, and low returns.

#### 3.1.2 Density gradient centrifugation

The density gradient centrifugation refers to the method in which components with different settling coefficients settle at a certain speed under the action of centrifugal force, forming bands in different density gradient regions. The PELNs obtained by UC method can improve purity under density gradient centrifugation, and sucrose and iodixanol are two commonly used media for density gradient centrifugation (7). But this method takes a long time and the instruments are expensive.

#### 3.1.3 Ultrafiltration

Ultrafiltration is a molecular weight based screening method that uses membrane filters with different pore sizes to separate PELNs of different sizes, with a generally separable molecular weight range of 10–600 kDa. The ultrafiltration process first uses a filter to remove larger impurities (cells, cell debris, apoptotic bodies) leaving behind a permeate rich in vesicles, and then uses an ultrafiltration membrane smaller than the target vesicle pore size to remove low molecular weight impurities (free proteins, pollutants) (78). Ultrafiltration method requires short time and low equipment requirements, and is often used in combination with other technologies as a supplement to UC or SEC. However, due to the squeezing effect, the ultrafiltration process may change the structure of PELNs, and the membrane may have problems such as clogging and contamination (28).

#### 3.1.4 Size exclusion chromatography

SEC is also a separation method based on molecular size. When the sample passes through a column packing, the larger particles are eluted along the pores with the mobile phase first and the smaller particles are eluted later according to the selection effect of fixed relative molecular diameters (24). PELNs with larger particle sizes generally flow out first, while impurity proteins with smaller particle sizes stay in the chromatographic column for longer periods of time. The advantages of this method are high efficiency, easy operation, and time-saving. The obtained nano components can maintain their intact structure and biological activity, and the purity of the separated PELNs is higher (29). However, it requires specialized equipment and additional concentration steps to increase the extraction concentration.

#### 3.1.5 Polymer precipitation

The polymer precipitation is a scalable and efficient approach that reduces the solubility of PELNs through the interaction between water-soluble, micro amphiphilic polymer molecules and water molecules, forming a hydrophobic microenvironment to achieve separation. Usually, studies use polyethylene glycol (PEG) with a molecular weight of 6–20 kDa to co incubate with the sample overnight at 4 ℃, and then collect the precipitate by low-speed centrifugation (30). Compared with the ultracentrifugation method, the polymer precipitation method has a faster speed, relatively lower cost, and does not require special equipment. Currently, there are reagent kits available. However, these polymers may cause co precipitation of other substances such as nucleic acids, lipoproteins, or other proteins, reducing purity (31).

#### 3.1.6 Immunoaffinity capture-based technique

Immunoaffinity capture-based technique is currently one of the methods used for isolating and purifying specific classes of PELNs (32). This technology utilizes antibodies to capture specific PELNs with different protein labels on their surfaces, and then binds them to magnetic beads or other separation substrates through covalent or high affinity interactions. Subsequently, PELNs are separated and purified using low-speed centrifugation or magnetic techniques (33, 34). Immunoaffinity capture technology can achieve precise separation and effective enrichment of PELNs, but this method is costly, requires strict usage conditions, and is not suitable for a large number of samples.

#### 3.1.7 Microfluidics technology

Microfluidics technology is an emerging technique for separating PELNs in recent years. Microfluidics technology can distinguish, capture, enrich, and separate PELNs with very similar shapes and sizes. The sample only needs to flow through a small microfluidic device containing nanofilters, nanoporous membranes, or nanoarrays (35), and purify PELNs based on physical characteristics such as size, density, and surface antigens (36). Microfluidics technology can integrate sample processing, analysis, detection, and other processes into chips, achieving miniaturization, integration, high-throughput, and low sample volume (37, 38). Based on these advantages, microfluidics technology has gradually become a powerful tool for efficient separation of PELNs. When the sample is uniformly injected, PELNs will adhere to the inner surface of the channel and then be separated by immunoaffinity, mesh screening, etc (39). This separation method is fast and can achieve efficient separation of PELNs. At present, microfluidics technology has not been standardized, and research on the separation of PELNs is insufficient. However, it has great prospects in high-throughput analysis of PELNs in the future (40).

PELNs isolation faces distinct challenges due to contaminating cellular debris, proteins, and polysaccharides that compromise purity and yield.Current limitations in extraction efficiency and purity represent a major obstacle for standardizing and clinically translating PELNs. Therefore, it is imperative to develop more efficient and stable extraction methods for PELNs in the future.

### 3.2 Identification of PELNs

The identification methods for the three-dimensional morphology of PELNs include scanning electron microscopy (SEM), transmission electron microscopy (TEM), cryo electron microscopy (Cyro EM), and atomic force microscopy (AFM), which have the advantages of continuously adjustable magnification, large field of view, and stereoscopic imaging; At present, the methods reported in the literature for measuring the particle size of PELNs mainly include dynamic light scattering (DLS), nanoparticle tracking analysis technology (NTA) (41), and adjustable resistance pulse sensing technology (42); In addition to physical characterization, identifying biochemical features such as lipids, proteins, and nucleic acid omics is also a method for characterizing PELNs. Western Blot detection mainly detects marker proteins on the surface of extracellular vesicles. The detection indicators for animal derived extracellular vesicles are usually CD63, Tsg101, CD9, CD81, and HSP90, while the study of marker proteins for PELNs is not yet fully understood (43); Extensive protein analysis of extracellular vesicles was performed using liquid chromatography tandem mass spectrometry (LC-MS/MS) technology, and compared with known PELNs proteins (44). At present, the identification of PELNs mainly relies on electron microscopy observation of morphology and dynamic light scattering analysis of particle size distribution. There are already labeled proteins that can be identified in animal exosomes, but research on PELNs in this area is not yet mature.

## 4 Storage and stability of PELNs

### 4.1 Stability

Accumulated research has examined the effects of temperature, pH, simulated physiological environment, and ultrasound treatment on stability. Chen et al. (45) confirmed that storing fresh ginger slices or freshly isolated ELN at −80 ℃ did not significantly affect the IL-1β-inhibiting activity of ELN derived from ginger. Kocholata et al. (46) found that PELNs remain stable for approximately one year when stored at −80 ℃, whereas storage at −20 ℃ maintains stability for up to three months. Leng et al. (47) found that the optimal storage temperatures for blueberry-derived EVs were 4 ℃ for short-term storage and −80 ℃ for long-term storage. Storage at 4 ℃ helped to prevent ice crystals from damaging the phospholipid bilayer membranes of the PELNs, while storage at −80 ℃ slowed down the rate of degradation and maintained the particle morphology.Richter et al. (48) found that PELNs stored at −80 ℃ and 4 ℃ had higher particle recovery than freeze-dried PELNs. However, through the research of Rehmania derived nanovesicles (RDNVs) at different storage temperatures and storage times, it was found that temperature alone may not be sufficient in safeguarding the activity and stability of RDNVs (49). Similar results have been confirmed in many plant derived ELNs, but little in-depth research has been conducted (50, 51). Monitoring the stability of ELNs under different pH values and simulated gastrointestinal environments is necessary for oral administration. It is worth noting that the lipid bilayer membrane structure present on the surface of plant ELNs exhibits remarkable resilience against the harsh conditions imposed by gastric acid and bile (52). Chen et al. (39) reported that the particle size and zeta potential of Camellia sinensis ELNs were unchanged in gastric, small intestinal, and colonic fluids. In contrast, the surface charge and electrostatic repulsion of PELNs with negative charge could be reduced in the stomach acidic environment, resulting in aggregation of vesicles.values and simulated gastrointestinal environments is necessary for oral administration (53). The particle size of ginger ELNs decreased from 243 nm to 228 nm after incubation for 30 min in simulated gastric fluid and to 216 nm in simulated intestinal fluid, and the zeta potential was weakly positively charged in the acidic environment of simulated gastric fluid (0.26 mV) and negatively charged in the neutral environment and simulated intestinal fluid (−14.2 mV and −7.3 mV), confirming that ginger ELNs can change their physical properties according to the environment to ensure that they are not degraded (54). Although the particle size of yam ELNs changed, the number of vesicles did not change, and they could still play corresponding targeting and communication roles (55). These studies evaluated the changes in the morphology and size of PELNs in storage media with different pH values, and none of them showed significant changes and maintained a relatively stable structure.

### 4.2 Storage methods

The simplest method to preserve plant samples is natural or artificial drying. Many fresh herbs need to be dried before storage or sale. Woith et al. (56) reconstructed tissue cells by soaking tobacco (Nicotiana tabcum L), Vinca minor L, and Chinese cabbage (Viscum album L) in a buffer solution for 24 h, and subsequently isolated EVs with particle size and morphology consistent with fresh plants. This can provide a simple method for preserving PELNs.

PELNs are mostly prepared and used on-site, and systematic research on preservation methods is still limited. In the field of mammalian derived EVs, several preservation methods have been developed and tested, including cryopreservation, freeze drying, spray drying, etc (57).

## 5 Functions and applications of PELNs

### 5.1 Participating in intercellular communication in plants

PELNs may maintain cell structure and function by secreting cell wall related proteins, as well as clearing harmful products from cells and participating in immune surveillance processes. The proteomic analysis of sunflower seed vesicles showed that PELNs are related to the secretion of enzymes that modify the cell wall (58). In addition, PELNs can also participate in cell proliferation, differentiation, and response to stimuli such as stress (59). Research has shown that PELNs are widely involved in self-defense responses, especially playing an important role in inducible defense mechanisms (60). Cai et al. (61) found that Arabidopsis thaliana secretes PDVs from the infected site when infected with Staphylococcus aureus, and introduces the carried sRNA into the fungal body, silencing key pathogenic genes and playing a cross species defense regulatory role. Rutter et al. (19) found that the secretion of PELNs in Arabidopsis infected with Pseudomonas syringae increased. Similarly, the secretion of PELNs also increased after treatment with salicylic acid, indicating that PELNs are involved in the plant's immune defense response.

### 5.2 Whitening effect

Lee et al. (62) found that the stem and leaf derived vesicles of Huangqi wood have anti melanin production effects. Applying vesicles derived from the stems and leaves of Huangqi wood to B16BL6 melanoma cells can significantly reduce melanin content and tyrosinase (TYR) activity, while leaf derived vesicles can inhibit the expression of melanin producing genes and enzymes. Its whitening activity is superior to the positive control group of arbutin, and there is no significant toxicity. Similar results have also been confirmed in the human epidermal model.

### 5.3 Treatment of inflammatory diseases

In the past few decades, research has shown that diets, especially dietary bioactive ingredients, play a crucial role in improving human health and intervening in disease development. Research has shown that PELNs can regulate inflammatory pathways by interacting with chemokines, interleukins, and other factors. Scallion and garlic PELNs inhibit the generation of NLRP3 inflammasomes in a dose-dependent manner, reducing the mortality of macrophages and eosinophils (63). Dietary PELNs, when in contact with the gastrointestinal tract, can enhance the integrity of the intestinal barrier through various mechanisms such as regulating gut microbiota, modifying intestinal epithelial cells, and improving the intestinal immune system. Research has shown that bitter buckwheat PELNs can promote the growth of Escherichia coli and Lactobacillus rhamnosus, and increase the abundance of gut microbiota (64). Grape PELNs can help accelerate the normal renewal of gastrointestinal epithelial cells in subjects, and can also reduce the levels of cytokines IL-6, IL-1 β, inflammatory monocytes, and specific chemokines recruited by T cells in a mouse colitis model (79). PELNs can also penetrate the blood-brain barrier and reach the brain to exert their effects. Oat PELNs cross the blood-brain barrier through the free diffusion and active transport of endothelial cells, significantly reducing the levels of IL-6, IL-1 β, and TNF—*α* in the mouse brain, while inhibiting the infiltration of microglia and reducing the number of activated microglia (65).

### 5.4 Intervention effect on cancer

PELNs and extracellular vesicles have similar effects in the treatment of diseases such as cancer. Compared with mice treated with PBS alone, citrus PELNs can inhibit cell proliferation in different tumor cell lines by activating the expression of TRAIL/DR5 pro apoptotic molecules (66). Stanley et al. (67) proved that grapefruit PELNs can specifically inhibit the proliferation of lung cancer, skin cancer and breast cancer cells by reducing the expression of cyclin, intercellular adhesion molecule and cathepsin. In addition to inhibiting cell proliferation, PELNs can also promote tumor cell apoptosis. Ginseng PELNs can promote M1 like polarization of M2 macrophages, thereby increasing ROS production and promoting apoptosis of mouse melanoma cells (50).

### 5.5 As a drug carrier

In recent years, PELNs from fresh plants have been regarded as natural therapies and nanoplatforms for combating various human diseases. This type of particle, which is completely self-assembled by pure drugs or therapeutic ingredients, is also called a carrier free nanoplatform (68). At present, PELNs can be loaded with various drugs to treat brain tumors, colon cancer, breast cancer, oral cancer, melanoma and colon metastatic liver cancer. The blood-brain barrier in mammals hinders the action of central nervous system drugs, while PELNs can act through the blood-brain barrier. *in vitro* experiments have shown that PELNs can encapsulate functional miRNAs and deliver them to mouse glioma GL-26 cells, activating natural killer cells to treat mouse brain tumors (69). In colorectal cancer, cabbage PELNs loaded with doxorubicin can successfully inhibit the proliferation of colon cancer cells and enhance their targeting to colon cancer cells through folate modification (70). Li et al. (71) conducted an *in vitro* anti-cancer study on the encapsulation of astaxanthin in PELNs from broccoli and found that it enhanced the inhibitory effect of astaxanthin on human colon cancer HT-29 cells.

## 6 Challenges and perspectives

In recent years, PELNs have become a research hotspot in biology, medicine, and other fields. Many researchers have shown that PELNs are from edible plants or herbs, they are safe, reliable have little side effects, and play a great role, and biological functions are also recognized increasingly. PELNs carry protein, lipids, nucleic acids, metabolites, etc., which can promote relevant applied research. the physiological and pathological conditions from the cell sources, play an important role in substance exchange and information transmission between cells. Nowadays, the biological research on PELNs is still in the primary stage, and their biogenesis pathway, release and uptake mechanism, and signal transduction pathway need further study.

The safety of PELNs is a necessary prerequisite for extensive research and clinical translation. Although PELNs have shown no toxicity in most studies, intravenous injection in some studies has raised some concerns about safety, creating challenges for their applications. PELNs contain some complex undetected nucleic acids, proteins, and lipid metabolites (72), which are natural components that are less biohazardous (73). Chen et al. (74) showed that mice were injected with tea ELNs four times, the body weight of the mice decreased, and the mice were assayed for AST/ALT and urea nitrogen creatinine, which showed that the tea ELNs produced potential hepatorenal toxicity and led to alterations in the blood picture, as well as an increase in the concentration of inflammatory factor TNF-alpha and complement C3 compared to the healthy group. Cong et al. (75) showed that Rehmanniae radix ELNs exhibited significant cytotoxicity after overnight storage at −80 ℃ in coculture with RAW 264.7 cells, but the reason for this remains unclear. At the same time, the immunogenicity or variability of different PELNs are uncertain, which still need further basic experiments for research and verification. Whether all types of PELNs can reach target cells without any toxicity to exert possible biological effects still needs to be further explored, and the concentration and purity of PELNs that can be isolated from different types and genera of plants are issues that must be considered. There are still many unknown areas waiting to be explored.

The standardization of preparation and scaling-up processes for PELNs is crucial for their industrialization and commercialization. At the laboratory level, researchers have optimized the isolation methods for PELNs to enhance yield and reproducibility, and have validated their biological activity (76). However, systematically integrating these research findings into existing pharmaceutical, agricultural, and cosmetic manufacturing frameworks remains a critical challenge. Moreover, standardization during the production process is another significant challenge. Differences in plant sources, growth conditions, and preparation methods can result in variations in the structure and functions of PELNs (77). Therefore, establishing standardized production and quality control systems are crucial for the successful large-scale deployment of PELNs. Standardized protocols ensure the reproducibility and reliability of ELNs products.

## 7 Conclusion

In recent years, plant-derived exosome-like nanoparticles have become a research hotspot in fields such as biology and medicine, especially the study of edible PELNs is constantly increasing. However, there is still limited research on PELNs. The main problems in the research of PELNs are: firstly, the extraction methods of PELNs are relatively single, and there is also little research on the optimization of extraction processes; Secondly, animal exosomes can be identified by labeling proteins, but the most commonly used methods for identifying PELNs are still electron microscopy observation and particle size analysis; Thirdly, the research objects of PELNs are more like extracellular vesicle nanoparticles, not entirely extracellular vesicles, with a wider range. If the extraction process and identification methods can be further optimized, the study of PELNs will be more convincing. Previous studies have shown that PELNs play a certain role as therapeutic agents, drug carriers, and other aspects, indicating that the development prospects of PELNs are very broad in these areas.
